# Child psychiatry expertise in the context of parental separation

**DOI:** 10.1192/j.eurpsy.2021.1683

**Published:** 2021-08-13

**Authors:** M. Regaya, A. Guedria, T. Brahim, N. Gaddour, L. Gaha

**Affiliations:** 1 Department Of Psychiatry, University of Monastir, Faculty of Medicine of Monastir, LR05ES10, Fattouma Bourguiba Hospital, Monastir, Tunisia; 2 Child And Adolescent Psychiatry Department, Fattouma Bourguiba University Hospital, University of Monastir, Monastir, Tunisia

**Keywords:** Child Psychiatry, expertise, parental separation

## Abstract

**Introduction:**

Marriages’ dissolution phenomenon had increased in recent years in Tunisia. The impact of divorce on children depends on the interweaving of several factors and is not inevitably pathological. We have noticed in our daily practice a concomitant increase in the number of request for expert opinions concerning children.

**Objectives:**

Determine the clinical children’s profile of separated parents carried out within the framework of legal expertise.

**Methods:**

We carried out a retrospective study in the outpatient child psychiatry ward at Fattouma Bourguiba general hospital in Monastir, Tunisia. Including all the expert reports of children affected by parental separations during a period of two years (2017 to 2019).

**Results:**

56 children were included in our study. The average age were (6.7 years) with a majority of males (58.2%). School failure concerned (24%). In most cases, the request for expertise was made in the context of mistreatment’s suspicion (60.7%), than following the parents’ separation (16.1%). Concerning the clinical picture: a normal psychiatric examination was found in the majority of cases (55.4%), anxiety symptoms concerned (32.1%). Cases of depression, global developmental delay and autism were also found.

**Conclusions:**

According to our study, the vast majority of children presented a normal psychiatric examination. Moreover, a preponderant part of the symptoms seemed to result from educational errors. While parental separation poses risks for children, research shows that these negative effects are not the same for everyone. Several factors can reduce these risks and promote children’s resilience. Thus, first-line psychosocial care should be offered for families and children in seprations’ context.

**Disclosure:**

No significant relationships.

